# Facing new challenges to informed consent processes in the context of translational research: the case in CARPEM consortium

**DOI:** 10.1186/s12910-021-00592-9

**Published:** 2021-03-02

**Authors:** Elise Jacquier, Pierre Laurent-Puig, Cécile Badoual, Anita Burgun, Marie-France Mamzer

**Affiliations:** 1 Centre de Recherche Des Cordeliers (UMRS 1138), INSERM, Sorbonne Université, Université de Paris, Team ETREs, 75006 Paris, France; 2 Centre de Recherche Des Cordeliers (UMRS 1138), Team Personalized Medicine, INSERM, Sorbonne Université, Université de Paris, Pharmacogenomics and Therapeutic Optimization, 75006 Paris, France; 3 Pharmacogénétique Et Oncologie Moléculaire, Hôpital Européen Georges Pompidou, Assistance publique – Hôpitaux de Paris, Paris, France; 4 Centre de Ressources Biologiques, Service d’anatomo-pathologie, Hôpital Européen Georges Pompidou, Assistance publique – Hôpitaux de Paris, Paris, France; 5 Département D’informatique Médicale, de Biostatistique Et de Santé Publique, Hôpital Européen Georges Pompidou, Assistance publique – Hôpitaux de Paris, Paris, France; 6 UMR-S 1138, Centre de Recherche Des Cordeliers, Paris, France; 7 Faculté de Médecine, Université Paris Descartes, Sorbonne Universités, Paris, France; 8 Unité Fonctionnelle D’éthique Et Médecine Légale, Hôpital Necker-Enfants Maladies, Paris, France

**Keywords:** Dynamic consent, Informed consent, Translational research, Biobank research, Patient participation, Partnership in research

## Abstract

**Background:**

In the context of translational research, researchers have increasingly been using biological samples and data in fundamental research phases. To explore informed consent practices, we conducted a retrospective study on informed consent documents that were used for CARPEM’s translational research programs. This review focused on detailing their form, their informational content, and the adequacy of these documents with the international ethical principles and participants’ rights.

**Methods:**

Informed consent forms (ICFs) were collected from CARPEM investigators. A content analysis focused on information related to biological samples and data treatment (context of sampling and collect, aims, reuse, consent renewal), including the type of consent. An automatic assessment of the readability of the ICFs were performed with the IT program “Flesch Score”.

**Results:**

29 ICFs from 25 of 49 studies were analyzed after selection criteria were applied. Three types of consent were identified: 11 broad consents, six specific consents, and two opt-out consents. The Flesch Scores showed that most of the documents were too complex to be fully understood by most of the potential research participants. Most of the biological samples were collected during the healthcare routine, but the information content about secondary use of biological samples varied between ICFs. All documents mentioned personal data treatment but information about their reuse was not standardized in the ICFs.

**Conclusions:**

Our review of current IC procedures of CARPEM showed that practices could be improved considering new translational research methods. “Old fashion written ICFs” should be adapted to the translational research approach, to better respect individual rights and international research ethics principles. In this context, theoretically, a digital tool allowing dynamic information and consent of participants, through an electronic interactive platform may be a good way to promote more active participation in research. Nevertheless, its feasibility in the complex environment of biological samples and data research remains to prove. The way of a combination of a broad consent followed by dynamic information may be alternatively tested.

## Background

Translational research in health can be defined as a multidisciplinary approach that gathers several areas of scientific expertise to achieve a common concrete goal through knowledge transfer [1]. In other words, a continuum between different scientific areas including different knowledge sets, methodologies, and findings should result in translating research into practice [2]. Hostiuc et al.characterized translational research in the context of the European Union’s Horizon 2020 Program to get a consensus on the definition and characteristics of the translational research program in different contexts [3, 4]. In healthcare, translational research has been recently described as a process that starts “from the bedside, goes to the bench of scientists, and comes back to the bedside” [5]: patients donate their biological samples and data for research, scientists use them for various types of basic science research, and the research findings can result in new clinical therapeutics and new healthcare policies for patients [3, 6]. The way that cancer research is carried out has profoundly changed because of this scientific global approach: every scientific step is part of the translational approach, from basic to clinical research and outcomes research [7]. In that way, innovative studies are now dependent upon infrastructures, such as biobanks and data repositories, in which biological samples and healthcare data are collected and can be used for various *ex-vivo* non-interventional phases of translational research. The duration of the storage should better be unlimited and sharing data and samples at a local, national, or international level has become essential [5, 8–10]. In this specific context, biological samples and data are at the heart of the health research. The principles of the Declaration of Helsinki were reviewed in the Declaration of Taipei to apply to the scientific use of biological samples [11, 12]. Besides, as biological samples are valuable only if they are coupled with associated data, they raise similar issues to those concerning data treatment [13].

This new organization raises some ethical issues linked to information and consent practices. The duty for researchers to obtain informed consent from potential participants before the beginning of any research project is a fundamental principle of medical research, enshrined in the Declaration of Helsinki as well as in the General Data Protection Regulation (GDPR). The GDPR defines informed consent as “a clear affirmative act establishing a freely given, specific, informed and unambiguous indication of the data subject’s agreement to the processing of personal data relating to him or her, such as by a written statement, including by electronic means, or an oral statement” (Considerant 32) [14]. Informed consent is a way to ensure that patient autonomy is respected, which is a key principle of medical research [15, 16]. It should attest that each research subject has received adequate information about the study either verbally or written. Investigators have to provide proof that fair information has been delivered to comply with the law if they want to conduct research and publish their findings. The French law provides also that informing someone and obtaining their consent to participate in research or use biological samples or data for research purposes has become a legal obligation [17–19]. Nevertheless, new methods of translational research are challenging traditional ways of asking informed consent. In this context, the literature describes three main types of consent forms (specific consent, broad and opt-out consent) [20, 21]. The specific consent form provides to participants precise and specific information about the objectives of the biological samples and data use (for health purpose or a single study for example), but it implies that these biological resources will be unavailable for other scientific purposes. The broad consent form promotes scientific freedom while participants consent once and for all whatever the circumstances of the initial storage (health care or research purposes) [22–24]. It satisfies as closely as possible translational research expectations by allowing the unlimited duration of the storage and sharing of samples and data at a local, national, or international level [23, 25]. The opt-out consent form does not require the explicit consent of the participant, but it obliges the practitioner to inform the patient: if s/he disagrees, the patient has to say his/her disagreement unequivocally [21]. This consent form is specifically used to allow researchers to use health data and biological samples collected during healthcare. The French Data Protection law, amended after the GDPR had come into effect, as well as specific international ethical principles, are challenging both broad and opt-out consents as they request specific information and if necessary ongoing information related to data processes to avoid that people involved in a research program lose the link with their biological samples and data [12, 14, 26].

Even though all these informed consent procedures have been well distinguished theoretically in the scientific literature, but it is unclear if they are sufficient or not to guarantee the patient’s rights to be informed and to consent freely. We hypothesized that investigating informed consent (IC) practices of a French expert research consortium could help to identify the ethical boundaries of these current IC procedures.

The Cancer Research for Personalized Medicine (CARPEM) SIRIC, accredited by the French National Institute of Cancer (INCa), is an expert consortium in translational research and precision medicine in the field of cancer. Precision medicine is an example of the conception of translational research: it uses fundamental and clinical research to create targeted therapies for patients whose tumors share common genetic characteristics. It also reuses data repositories to generate hypotheses, to adapt treatments, and to discover new indications for existing drugs [27, 28]. Since its creation, the CARPEM SIRIC has developed a translational research platform which integrates all the data (clinical, research, -omics, the existence of samples, etc.) from CARPEM patients into a unique data warehouse. This flagship platform is an essential transversal tool for all CARPEM members who can access data relevant to their study, and most CARPEM researchers and clinicians are involved in the warehouse and the development of data exploring tools. Furthermore, few collections of biological samples are created in the different CARPEM infrastructures, and they are gathered in a unique catalog that references all types of biological samples available for research. Biological samples came from either care or interventional research and can be reused for non-interventional studies. Interventional research qualifies a study that requires to do an invasive procedure on the participant, as a blood draw made especially for scientific purposes. Non-interventional research qualifies a study without any invasive procedure for scientific purposes: biological samples can be part of healthcare procedure and a part of it can be used for scientific purposes. Until now, the CARPEM system has adopted a dual solution to collect health data and biological samples, as required by French law [18]. Data collected during the care process are integrated under an opt-out consent policy, following the policy of the Assistance Publique – Hôpitaux de Paris (AP-HP) concerning their health data warehouse whereas when data are collected specifically for research purposes, an opt-in solution is legally imposed. Similarly, researchers can use healthcare biological samples for research purposes only if the patient had been informed and agreed with. According to French law, this informed consent is either an opt-out consent if healthcare biological samples are used without genetic research purposes, or an explicit consent if genetic research is planned [19, 29]. Indeed, even if the Declaration of Taipei and the European GDPR require explicit consent from the concerned individual, European state members remain responsible for the regulation of research, and some legal facilities are permitted.

The context of CARPEM provides the opportunity to analyse the practices related to information and consent in the translational research approach, from the time of care to the “phase 0” of collecting and using biological samples and data [3]. Indeed, the use of biological samples and personal data in research has been extended in two ways: first, collecting biological resources has become easier thanks to the link between healthcare and research; second, their use has been expanded thanks to the new role of specific infrastructure as biobanks and databases. In the light of these considerations and of ethical perspectives of transparency, respect for individuals and scientific expectations, we aimed to assess the characteristics of various informed consent forms used by CARPEM researchers at the moment they collect data and biological samples for their research purposes by reviewing the current IC process of CARPEM: what kind of information current IC forms provide to research participants and how could they be improved? Our review focused on the written content of IC forms and allow us to question the use of IT technology to improve information and the consent process.

## Methods

A qualitative analysis was performed to assess the quality and content of information of a set of informed consent forms that were used by CARPEM researchers between 2012 and 2017.

### Collecting informed consent documents

In October 2017, all CARPEM investigators listed in a 5-years activity report about the activities of CARPEM were asked by email to provide the informed consent forms from studies they had started between 2012 and 2017. New emails were sent to investigators who had not answered the first one at the beginning of November and December 2017. Investigators sent back ICFs by email voluntarily.

Because the study focused on information about biological samples and data treatment, only studies that planned to collect biological samples and data were selected among all received documents. Collected informed consent forms (ICFs), including information documents and consent forms, were studied to evaluate their readability using a specific and validated IT solution (the Flesch Score; see below) and to analyse information content delivered about the collection and the use of biological samples and data for research purposes [30]. The CARPEM 5-years activity report (unpublished data) and website https://clinicaltrials.gov/ct2/home were consulted to identify the types of research related to informed consent forms (non-interventional or interventional).

### Automatic evaluation of the readability of the documents with Flesch score

The IT program Flesch Score had been developed by R. Flesch to assess the readability level of texts thanks to an algorithm using the average sentence length and the average word length [31]. It has been adapted to be relevant for French written information and consent forms by Ménoni et al.[30]. As collected documents were written in French, the French version was used to evaluate the readability of the informed consent documents. Information documents and consent forms were submitted separately to estimate the readability level of each document to determine if information documents and consent forms had the same readability level or if they were different. The interpretation of the Flesch scores from 0 to 100 was performed according to the interpretation sheet available online [32]. A “very complex” document corresponds to a level higher than that of a university bachelor, a “complex” document to a university bachelor level, and a “quite complex” document to a high school level (Tables 1,2, 3).

**Table 1:** Codes used to perform the content analysis

Code	Description
Consent form	Consent forms determined the scope of their content and the choices someone can make when s/he signed it
New consent	Any information about renewing the initial consent
Right to withdraw	Any information about the right to withdraw the consent
Context of biological sampling	Were Biological samples collected during healthcare or during research?
Storage place	Any information about the place where biological samples were stored
Place of use	Any information about the place where biological samples would be used
Period of storage	Any information about how long biological would be used for scientific purposes
Purpose of use	Any information about scientific goal of using biological samples
Secondary use of biological samples	Any information about future use of biological samples after the first research
Transfer of biological samples	Any information about future transfer of biological samples, to private or public organization and/or to international structure
Right to ask for sample’s destruction or to oppose their reuse	Any information about the right to ask for sample’s destruction or to oppose their reuse for scientific purposes
Type of data	Any information about a type of collected data: biological, demographical, gender, genetic, medical record, lifestyle
Privacy	Any information about how privacy would be preserved: is privacy protection mentioned and is it explained?
Personal rights about data	Any information about personal rights on personal data: right to access and to change data, right to oppose to their use
Secondary use of personal data	Any information about potential secondary uses of data

**Table 2:** Details about selected studies and their corresponding information and consent forms

	Non-interventional study	Interventional study	Total
Number of studies	12	13	25
Number of consent forms	15	14	29
Broad consent	8	3	11
Specific consent	0	6	6
Broad or specific consent	5	5	10
Opt-out consent	2	0	2

**Table 3:** Flesch score interpretation sheet

Flesch score	Stylistic level	Grade level	Number of information documents	Number of consent forms	Total
0 to 30	Very complex	Academic level	10	12	22
30 to 50	Complex	Bachelor level	14	15	29
50 to 60	Quite complex	Highschool	0	2	2

### Data collection about biological samples and data treatment

Our analysis aimed to identify the written information content provided about data treatments and the scientific use of biological samples. After distinguishing the types of research (non-interventional or interventional), we focused on pieces of information about the collection and the use of biological samples and data for scientific purposes which were recorded in the documents. After a comprehensive overview, documents were read carefully to identify “codes”, “descriptive or conceptual labels” that qualify significant excerpts about biological samples utilization and data treatment. The codes were completed with legal provisions and helped to elaborate an analysis framework; a spreadsheet used as a matrix to summarized relevant data. Then, trough many readings, all relevant data were collected by applying the analysis framework and summarized into the matrix [33].

### Content analysis of informed consent forms

The content analysis focused on studies that included a collection phase and storage and use of biological samples and personal data for research purposes. Informed consent forms were characterized based on their content and the choices at the time of consent and classified into the following categories: “specific consent” (SC), “broad consent”(BC), “either broad or specific consent” (BSC), or “opt-out consent”(OC) [20]. In the context of the use of biospecimens for research, “specific consent” consisted of consent requested for specific research and “broad consent” for the “the collection and storage of biospecimen for future unspecified research, which will occur under conditions defined at the time of consent.” “Either broad or specific consent” allowed the subject to choose between the use of the specimen for specific research or also for future unspecified research. “Opt-out consent” means that people are informed of “the research use of biospecimen and are offered an opportunity to opt-out” [20].

The following specified information about the collection of biological samples was taken from the documents: the context of the collection (care or research), storage place, place of use, the period of storage, the purpose of use, whether reuse was considered, and whether the initial consent was planned to be renewed. If the transfer of any samples were considered, information with any mention of the place and context of transfer (partnership or disposal) were also collected. Information about the right to withdraw consent or to ask for the sample’s destruction was also verified. Care as a context of the collection refers to the clinical procedure during which samples were obtained for clinical purposes, but a part of them would also be used for research purposes. On the contrary, research as a context of the collection refers to the fact that samples are obtained during an additional procedure outside the scope of healthcare.

Finally, documents with information concerning data collection were analysed to determine what kind of data was collected (medical data, lifestyle data, genetic data, or data from biological samples), how privacy was protected, what personal rights were mentioned, and whether any reuse was planned.

This content analysis was performed to better define the limit of the use of biological samples and personal data for scientific purposes, thanks to informed consent. The personal rights related to biological samples and those related to personal data were considered separately because the French law distinguishes them: the GDPR regulates the rights related to personal data while the *Code de la Santé Publique* regulates the rights related to biological samples [14]. The results would be discussed considering legal provisions.

## Results

**Fig. 1:**
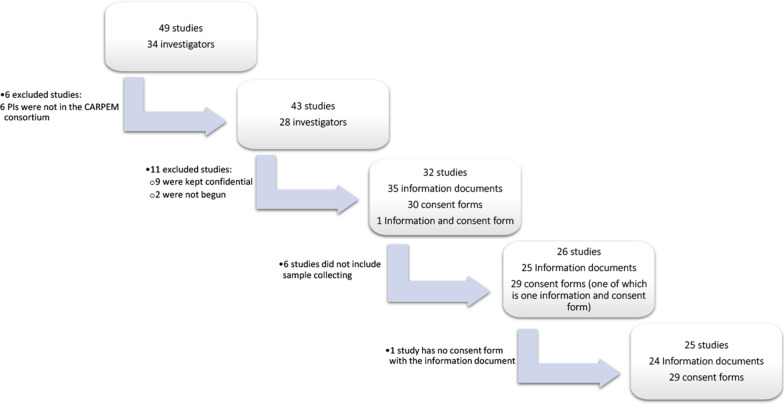
Flowchart

According to the CARPEM 5-year activity report (unpublished data), CARPEM investigators participated in 49 studies that started between 2012 and November 2017. Information and consent documents from 32 of 49 studies were gathered. Twenty-five out of those 32 studies planned to collect biological samples. Four studies required that a research participant signed two consent forms: one for the study and one specifically for genetic research. In all, 29 informed consent forms were analyzed, related to 25 studies. Informed consent forms associated information documents and consent forms, for a total of 53 documents (Fig. 1).

Overall, there were three types of consents: 11 broad consents (BC), 6 specific consents (SC), and 2 opt-out consents (OC). Opt-out consent forms were analyzed as information documents. Ten consent forms were either broad or specific consent (BSC). Informed consent for genetic research was either SC or BSC. Details about the studies selected for our survey and their corresponding information and consent forms are described in Table 2.

### *Evaluation of the readability of the documents *via* the Flesch score*

The Flesch scores of documents ranged from 14.7 to 54.2, according to the interpretation sheet [32]. Twenty-two of 53 documents were evaluated to be “very complex” (score from 0 to 30), 29 “complex” (score from 30 to 50), and two “quite complex” (score from 50 to 60) (Table 2).

### Information related to the use of biological samples for scientific purposes

**Fig. 2:**
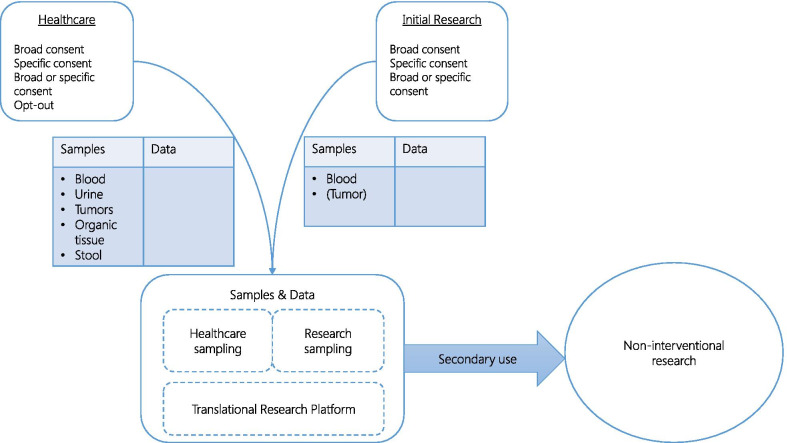
Biological samples and data process from the collection to the use in research

Most of the biological samples used in the studies came from healthcare (16 of 25). In all but one study, the tumor samples were healthcare samples. In one study, an additional sample had to be collected specifically for the research purpose if no healthcare tumor sample was available. In 4 non-interventional research studies, additional blood samples had to be collected during healthcare sampling (Fig. 2).

The duration of storage was mentioned in only nine of 25 studies. Among these, the duration of storage was specified as “unlimited” for three studies (one BC, one BSC, and one TC).

The three identified types of consent were used (broad, specific, either broad or specific) whatever the type of the study (either for non-interventional and interventional ones). In 2 cases, opt-out consent was used when biological samples came from healthcare. Eighteen out of 21 BC or BSC forms reminded patients of the right to opt-out or ask for the destruction of the biological samples (Fig. 2).

The nature of information about secondary use varied from one document to another (Table 4). Four documents (two BSC, one BC, and one SC) mentioned that consent would be requested if any reuse was planned: one SC form mentioned that consent would be asked again if any reuse was planned; and in three cases, although the forms were either BSC or BC, they planned to ask for new consent if the reuse project was too different from that of the initial consent.

**Table 4:** Number of consent forms and studies with information related to biological use, according to consent forms

	Number of studies	Number of consent forms	Large consent	Specific consent	Either large or specifi consent	Opt-out consent
Total of studies with samples collecting	25	29	11	6	10	2
Sampling from care	16	17	6	3	6	2
Sampling from research	4	6	2	3	1	0
Sampling from care and research	1	4	2	1	1	0
Mention of a reuse	19	22	8	1	11	2
Mention of future transfer	13	14	6	0	7	1
Mention of future international transfer	5	5	0	0	5	0
Mention of transfer to public or private organization	10	11	6	0	4	1
Right to ask for sample's destruction of to be opposed to the reuse	20	23	10	3	8	2

### Information related to personal data processing

All the studies planned to use personal data and all information documents mentioned it (Table 5). Personal data were mainly designed as “healthcare data extracted from medical records” (in 21 out of 25 studies; “data associated with the biological samples” (7/25); “genetic data” (5/25), or “lifestyle data” (8/25)), without any explanation.

**Table 5:** Number of consent forms and studies with information related to data treatment, according to consent forms

	Number of studies	Number of consent forms	Large consent	Specific consent	Large or specific consent	Opt-out consent
Total of studies with data collection	25	29	11	6	10	2
Nature of data	24	27	9	6	10	2
Data of medical fields	21	21	8	5	6	2
Data associated to biological samples	7	7	3	0	4	0
Data about lifestyle	8	8	3	2	1	2
Genetic Data	5	5	2	0	3	0
Guarantees of data privacy	26	29	11	6	10	2
Information about codification	19	21	6	5	8	2
Right to access and to change data	26	28	10	6	10	2
Right to be opposed to the use of data	26	29	11	6	10	2
Data reuse project	7	7	4	0	3	0

All documents referred to confidentiality and guaranteed the protection of privacy and when a method was described (21 documents, 19 studies), it consisted of codification. The CARPEM platform used the following method: a shallow de-identification algorithm has been applied to patient data since its creation. All directly identifying data, as defined by the HIPAA Safe Harbour Recommendation, are removed and a number between -365 and 365 is randomly chosen to shift all dates. Identifiable data (as defined by HIPAA) and clinical data are stored separately in databases installed on separate servers.

Finally, 7 of the 25 documents mentioned that data could be reused for future studies (four BC and three BSC).

## Discussion

### Informed consent process in the CARPEM consortium is put into question

#### The current information content is inadequate for translational research

The issues of information and the consent process in the “bedside to bench” phase of translational research are complex as they aim to [1] promote collaboration between patients, practitioners, and researchers while minimizing the number and invasiveness of research interventions and [2] help to regulate the use of biological samples by balancing the needs and expectations of researchers with the patients’ preferences [5, 34]. Indeed, to make the inclusion phase more efficient and attractive, and to make the research less time consuming after the inclusion phase, biological samples are collected increasingly during healthcare. In our study, 12 out of 25 studies were non-interventional studies, which means that patients sustained no specific invasive intervention for research purposes: biological samples were thus collected during healthcare, for healthcare purposes, and a portion of them have been qualified as research resources thanks to patient’s consent and IRB approval. By consenting, a patient permits researchers to use his (her) biological samples and data for research purposes in addition to healthcare purposes, without any additional constraint. Whereas healthcare and research practices are still claimed as separate ones worldwide, the frontier between them tends to become confused. Healthcare resources are “re-characterized” thanks to the informed consent process and the approval of an ethical committee [11, 35].

In this setting shouldn’t it be guaranteed that participants understand complex written documents? Our results about documents’ readability are consistent with previously published data [30, 36, 37]: based on interpretation of the Flesch score, all the analysed forms were classified as being too difficult to be understood by most of the population: informed consent documents were classified either into the categories « complex» or « very complex» [32]. This signifies that the required literacy levels required to understand them, correspond to the level of a high school or a bachelor’s degree. Thus, these documents do not fit with the literacy level of most of the population, based on available data from the studies of the French National Institute of Statistics and Economic (INSEE). In 2018, only 36,8% of the French population had a bachelor's degree [38], and 38,5% of the whole European population [39]. It makes that 22 out of 53 (42%) documents may be understood by less than 40% of the French population. Such a gap between the complexity of ICF and the population’s literacy level calls the validity of the informed consent into question.

Besides information content was quite uniform: they listed the pieces of information that should be included in the document, so they were partly compliant with the law. However, even if consent forms listed the pieces of information written in the law, many of them were not systematically written in the documents [40–42]. Information about individual rights related to personal data was the most presented in the IC forms, in a way to be compliant with the new European General Data Protection Regulation (GDPR). Information related to future use of biological samples or data was rarer than information related to personal rights. This observation puts into question the scope of the consent, especially in the case of broad consent: if the participant allowed biological resources’ reuse, s/he would not receive precise information about it because it could not be predicted at the time of consent. From a legal point of view, broad consent remains valid if all of the future purposes are consistent with the initial goal of the consent [19]. From an ethical point of view, ICFs aim to inform about the initial scientific goal and about the kind of future research purposes for which biological resources could be used. Yet, the lack of information about reuse shows that the scope of the consent remains unclear: according to our results, participants can’t know where or to whom their biological samples or their data would be transferred in the future.

Therefore, the compliance of information documents with the law and the validity of the consent are put into question.

### The current consent process is not suitable for translational research

In our study, research protocols were quite various, being either interventional or non-interventional, with specific consent, broad consent or opt-out consent (Table 1), but the way of obtaining consent remained the same: the consent form was signed only once. Only one study with specific consent mentioned that there would be another consent if researchers needed to use biological resources for a new study. In this way, the informed consent process appears formal and static.

The three identified types of consent and the combination of two of them seem not completely appropriate to address the expectations and rights of both researchers and research participants (broad consent, specific consent, either broad or specific consent, and opt-out consent). Broad consent is the most common form and seems to satisfy as closely as possible translational research expectations: 11 studies used broad consent form. More than that, for ten studies, broad or specific consent could be chosen depending on the checkmarks of the patient. This trend shows that informed consent should help to handle the feeling of scientific emergency and the contradictory injunction to produce relevant results in a short time while asking people to consent to specific studies [23, 25]. Indeed, on one hand, the cancer research community is all at once tempted and encouraged to share and reuse biological samples and health data in order both to promote scientific discovery and to enhance research producibility. But on the other hand, the duty of information has become a legal formality to obtain consent, with little regard for the quality and the accessibility of informational content.

Satisfying the duty of information prompts us to think about a more compliant and suitable IC procedure. Ongoing information seems adequate considering potential multiple uses of biological samples and personal data for scientific purposes. But currently, people involved in a research program lose the link with their biological samples and data. As they do not re-consent to any future research, they do not receive future information as they should have, especially if data are fully anonymized [22, 43].

### How to reorganize the informed consent process? Some lines of thought

#### Informed consent process needs to be boosted

According to the previous observations, it seems hard to guarantee that patients receive and understand information adequately and provide valid consent thanks to it [20]. To strengthen the research participants' capabilities while promoting research, the way for the new ethical concept of “dynamic consent” (DC) have been paved in the scientific literature. At first sight, DC offers a new approach to consent which is designed to meet the needs of current research practices and the preferences of participants [44, 45]. It was initially developed in the field of biobanking and viewed as a personalized and digital communication interface between researchers, patients, participants, and citizens, placing participants at the center of the decision process [46]. In practice, participants could stay regularly informed about research protocols requesting their biological resources and reconsent to these new protocols. They could also choose the consenting frequency by choosing between specific consent to each new research, broad consent to all research, or a specific field of research or renewing consent when there is a major change. Regular information on digital support is the key to this concept because participants can stay involved in research through information and their right to consent. A consensus between broad consent and specific consent seems to be affordable in response to our hybrid consent form (BSC): consent could become ongoing instead of one-off and could allow more flexibility for researchers and research participants. Research participant can change their mind depending on consecutive studies and researchers would have access to available biological resources according to consent changes. In this way, restoring the value of informed consent by using a digital interface as DC could promote the public acceptability of translational research methods by improving transparency of information and being more respectful of individuals.

From a legal point of view, the informed consent process would be more compliant with the new European Regulation for Data Protection (GDPR), which encourages this type of initiative by requiring specific consent for scientifically defined purposes and facilitating the collection of consent by IT tools. Although scientific research is an exception and informed consent can be less specific (Recital 33 and 159), the European Data Protection Board recommends updating the information to make consent as specific as possible [14, 47]. The previous boundary related to the compliance could be addressed thanks to this kind of IT tool.

Then, new trends in scientific data treatment bring not only data mining and analysis but also the integration of data from multiple sources thanks to more common and new technological information tools. In this way, linking information about the same individuals from different data sources (administrative, healthcare pathway, environmental, self-reported, or self-produced by connected objects) can be very useful, as the knowledge gained can be considered to be dependent on both the quantity and quality of the shared data [48]. In that case, ongoing informed consent would preserve participants' control over their data and maintain confidence in the scientific community which uses more and more personal data for research [49–51]. Keeping control of personal data may justify maintaining a link between individuals and their data. Moreover, it is difficult and even impossible to guarantee that data are completely anonymous, especially in cases of potentially identifying data, such as genetic ones [52]. The GDPR only considers data to be anonymous if the data subject cannot be identified by any means “reasonably likely to be used (…) either by the controller or by any other person” (Recital 23) [14]. Thus, even if a user of a database is neither able nor willing to re-identify a data subject, such a data set may still fall under the GDPR guidelines if it could be re-identified with reasonable effort. As a consequence, mechanisms for the assessment of the impact on privacy, pseudonymization and anonymization of data, fine-grained restriction of access, and requiring the use of formal agreements (Data Sharing Agreements, Data Re-use Agreements, Data Transfer Agreements) have grown in importance.

Dynamic consent could be another mechanism to control data access that comes from the data subjects [53]. People should be informed of the unphysical risks inherent to any research using biological samples and data. Yet, in our study, information related to the data security was not consistent and research participants barely knew what kind of data were processed for research purposes. But allowing research participants to keep control over their data by being able to renew their consent for data use may be an additional measure to preserve the public trust in research: participants may know who would use their data and for what purposes, and they could oppose furthering data treatment. It promotes the respect of GDPR's principle of transparency [49]. Digital technology can make this consent continuity and control over data happen if the scientific community commits to publishing information content regularly on such an online platform.

DC should aim at promoting the participant's autonomy by allowing them to renew his/her consent often [54, 55]. But reconsenting can block the research process because it is currently both time consuming and expensive. Moreover, many obstacles like consent fatigue and the risk of high drop-out rates can be disadvantageous for researchers. According to critics, DC could reproduce the same obstacles as SC [24, 49].

### Toward dynamic information of research participants

The main issue seems to be more the information content than the consent itself: qualitative studies highlighted the interest for regular information about secondary use of biological samples and personal data [50]. In that sense, people are not annoyed to consent only once, but they would prefer to be informed regularly about the use of their biological samples and personal data if they are looking for it [56–58]. Ongoing information looks like a priority to build and maintain public trust in non-interventional research through the transparency of information [25].

In the concept of DC, consent becomes dynamic because information could be ongoing over time and studies. It looks more like a process of communication in constant motion or change, depending on participants’ preferences [59]. First, informed consent is conceived as an ongoing process because the information is updated following the succeeded studies. Second, participants have access to information as much as they want on a dedicated platform. Third, they can alter their consent preferences according to their opinion about future study, by exercising their right of withdrawal. But this model needs to be sustained by a strong and regularly updated information content to guarantee consent validity. In our opinion, digital and ongoing information could complement the idea of dynamic consent, focusing on information content instead of the consent frequency.

Digital information would allow providing informational content through diverse supports, such as videos, or images on a smartphone, computer, or touchpad. Digital solutions can allow users to verify their understanding before giving their consent, like through quiz to succeed before consenting [60]. This would help to improve understanding because similar content would be provided differently and be respectful of individual preferences and capacities: for example, some specific content could be intended to the young public. Public specific informational content can be provided thanks to written texts but the concept of dynamic consent assumes that digital tools could make the communication easier [61]. Besides, accessibility to information requires that people can read in native languages: web support allows the possibility to have the same content translated into different languages [37, 62].

Another key factor of valid consent is the quality of information. It should strengthen the capabilities of research participants by strengthening their literacy level and helping them to decide whether they want to participate in research or not [37]. Health literacy is “the cognitive and social skills which determine the motivation and ability of individuals to gain access to, understand and use information in ways which promote and maintain good health” [36]. Information plays a large part in the informed consent process because it sustains the individual ability to decide freely and autonomously, only if it fits the health literacy level of the participant [63, 64]. However, our study showed that information was too complex to expect that most participants to biological samples and data research understood it.

A tool like a DC platform can be innovative support of information that aims to facilitate access and diffusion of informational content. It can go further by creating a direct but digital relationship between any participant and manager of biological samples and data or research teams aiming to use their biological material and thus, the participant could assert their interests and autonomy and be more confident with the scientific community [13, 50, 63]. Indeed, the interface should allow two-way communication to favor the creation of a more engaged participant population, in which research subjects can keep themselves informed. It can be customized to favor multiple interactions, including the implementation of self-reported data by patients. [45, 65, 66] Besides, if undefined future studies remained at the time of initial consent, participants could be informed later and over the studies. But now, one of the main issues remains as to who would be in charge of the task of building this platform of centralized information about scientific uses of biological samples and data. The stakeholder would be an intermediary between the research participant and researchers, like a biobank infrastructure whose mission is to collect, prepare to make available biological samples and data for scientific purposes [67]. In the CARPEM, as a major stakeholder in cancer research in France, the “Program 3: Dynamic consent and health democracy in CARPEM” has been aiming to conceive a DC platform with the support of the members of the CARPEM Patient Committee created during the first period of the CARPEM who would help to design the platform, guarantee the readability of information and rephrase it any time it would be required [5, 68].

### Limitations of the study

Our study has some limitations. First, the corpus of IC documents is quite limited because about a third of those used by the CARPEM’s researchers were not available for the study. Second, the survey focuses on the IC practices of a unique French research consortium, which does not depict all IC practices in France.

## Conclusion

We reviewed the current IC process of the CARPEM consortium regarding the new scientific standards of translational research in health that the consortium has adopted a couple of years ago. This review helped to show that some aspects of the IC process could be improved. Our survey highlights that the current translational research approach may favor the emergence of new ethical issues concerning informed consent practices. Indeed, in this context researchers increasingly use biological samples and data collected during healthcare to perform research while minimizing costs and risks directly linked to research protocols. Such practices may be accompanied by a new informed consent process to respect the ethical right of the individuals to decide whether researchers can use their material for secondary uses. Our qualitative study highlights part of the complexity of the underpinned issues: current information documents may be too complex to comply with the literacy level of a large part of the population, even when they are dedicated only to collect a specific consent and whereas their content is not thought to inform patients about the multiple but often still undefined further uses of their samples and data.

Besides, biological samples and data may be continually collected and stored for healthcare as well as for current and future unspecified studies. The increasingly frequent recharacterization of biological samples from healthcare to scientific purposes may encourage healthcare providers and researchers to adopt accountable management of biological samples for scientific purposes. It may also promote the organization of fair hospital biobanking for patient care while allowing the secondary use of the biological material for research purposes. Given these changes in health research practices, ethical standards updated by GDPR enforcement in Europe may be a way to reconceptualize and reinvent the practice of informed consent, especially because secondary uses cannot be anticipated at the time of consent.

IT tools could be useful for both researchers and individuals who consent to the use of their biological samples and personal data for scientific purposes. The “dynamic consent” concept is an example of such an IT tool that rests on the use of IT by research participants. Nevertheless, considering the complexity to deal with both information communication and consent management to remain compliant with all of the legal requirements, our next concerns will be to focus on the feasibility of better practices of information communication on the one hand, and better consent management on the other hand. Perhaps a combination of a first step consisting of global information followed by a broad consent, followed by dynamic information accessible through an electronic platform could be interesting.

## Data Availability

Informed consent documents can not be published because they are not anonymous. However, they can be consulted on request with the CARPEM director’s authorization, because they were not considered as confidential documents.

## Abbreviations

INCa: Institut National du Cancer
SIRIC: The Integrated Cancer Research Site
CARPEM: Cancer Research of Personalized Medicine
ICF: Informed Consent Form
BC: Broad Consent
OC: Opt-out Consent
BSC: Broad or Specific Consent
SC: Specific Consent
DC: Dynamic Consent
